# Construction of a prediction model for drug removal rate in hemodialysis based on chemical structures

**DOI:** 10.1007/s11030-021-10348-7

**Published:** 2022-01-01

**Authors:** Kousuke Nishikiori, Kentaro Tanaka, Yoshihiro Uesawa

**Affiliations:** 1 Department of Medical Molecular Informatics, Meiji Pharmaceutical University, 2-522-1Kiyose-shi, Tokyo, Noshio 204-858 Japan; 2 Meiji Pharmaceutical University Pharmacy, 1-3-6 Glanz Bldg 1F Higashikurume-si, TokyoHontyou, 203-0053 Japan; 3 Higashikurume Ekimae Clinic, 1-3-6 Glanz Bldg 2F Higashikurume-si, TokyoHontyou, 203-0053 Japan

**Keywords:** Quantitative structure–activity relationship (QSAR) analysis, Partial least squares (PLS) regression analysis, Artificial neural networks (ANN), Hemodialysis, Drug removal rate

## Abstract

**Abstract:**

In designing drug dosing for hemodialysis patients, the removal rate (RR) of the drug by hemodialysis is important. However, acquiring the RR is difficult, and there is a need for an estimation method that can be used in clinical settings. In this study, the RR predictive model was constructed using the RR of known drugs by quantitative structure–activity relationship (QSAR) analysis. Drugs were divided into a model construction drug set (75%) and a model validation drug set (25%). The RR was collected from 143 medicines. The objective variable (RR) and chemical structural characteristics (descriptors) of the drug (explanatory variable) were used to construct a prediction model using partial least squares (PLS) regression and artificial neural network (ANN) analyses. The determination coefficients in the PLS and ANN methods were 0.586 and 0.721 for the model validation drug set, respectively. QSAR analysis successfully constructed dialysis RR prediction models that were comparable or superior to those using pharmacokinetic parameters. Considering that the RR dataset contains potential errors, we believe that this study has achieved the most reliable RR prediction accuracy currently available. These predictive RR models can be achieved using only the chemical structure of the drug. This model is expected to be applied at the time of hemodialysis.

**Graphic Abstract:**

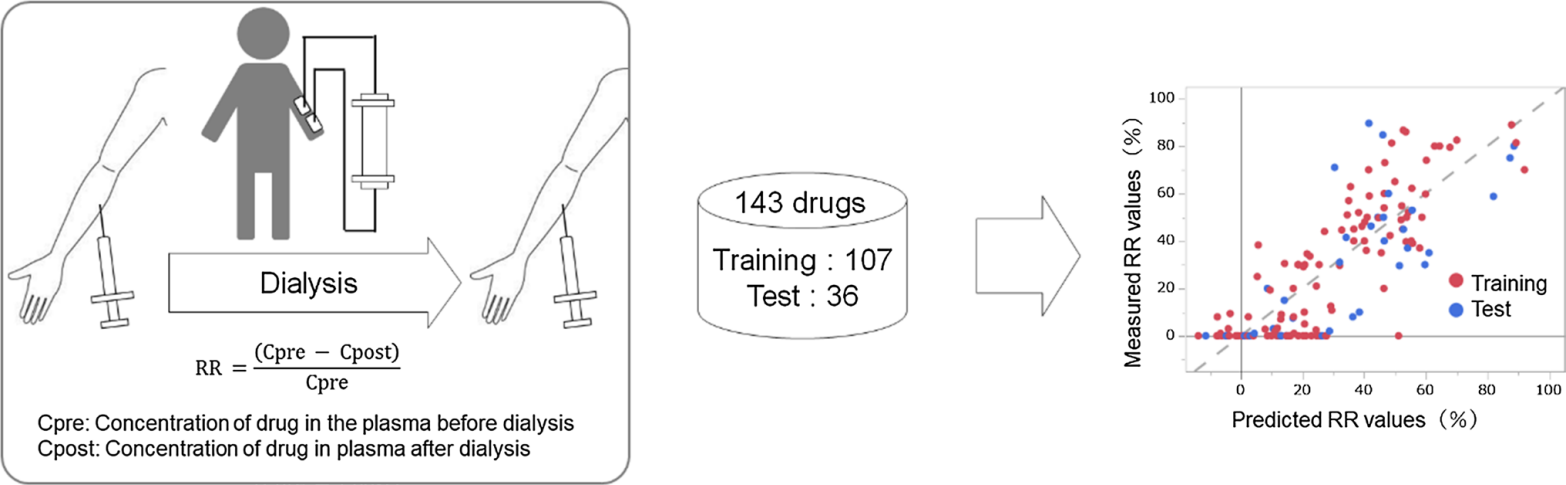

**Supplementary Information:**

The online version contains supplementary material available at 10.1007/s11030-021-10348-7.

## Introduction

When administering a drug to dialysis patients, it is necessary to replenish the amount removed by hemodialysis. To provide proper drug treatment for dialysis patients, it is important to determine the amount of drug replenished after hemodialysis. It can be calculated from the removal rate (RR) of the drug by hemodialysis. However, known RRs are limited to drugs that have already been reported, and the submission of RR data at the time of a new drug application is not required. Determining the RR is a clinical requirement; therefore, the estimation of RR represents an important technical problem. The RR is associated with pharmacokinetic parameters, such as the volume of distribution (Vd) and protein binding rate (PBR) of the drug [1], which are also known to be useful for the estimation of RR [2].

Furthermore, pharmacokinetic parameters are associated with physicochemical characteristics from the chemical structure, such as molecular weight (MW) and lipophilicity [3, 4]. In general, drugs with similar chemical structures have similar bioactive and physical properties. Quantitative structure–activity relationship (QSAR) analysis is a known method for modeling the relationship between chemical structure and efficacy using this principle [5, 6]. Information, in the form of “descriptors,” is used as explanatory variables to describe structural, physicochemical, and quantum chemical characteristics. QSAR analysis is a useful approach for modeling the correlations between physiological activity and various descriptors and for predicting RR. A prediction model constructed based on QSAR analysis may estimate the behavior of the drug in the human body without the need for a biological experimental system. In this modeling method, multicollinearity problems can be avoided using partial least squares regression (PLS) analysis combined with classical multiple regression analysis as a linear method [7, 8]. Recently, machine-learning methods have been applied to such analyses due to the high prediction performance of artificial neural networks (ANN) [9, 10].

Urata et al. [11] constructed an RR prediction model by multiple regression analysis using the pharmacokinetic parameters of MW, Vd, PBR, and a fraction of the unchanged drug excreted into the urine (fe). Moreover, other studies have reported the prediction of Vd and PBR using the QSAR approach based on the chemical structure [4, 12]. The chemical structure is an important predictive factor for RR. Based on these factors, QSAR analysis provides a high possibility of predicting the RR. In this study, the prediction of RR using QSAR analysis was performed using only the chemical structure. Therefore, its ability to respond to information regarding the drug’s pharmacokinetic parameters is limited. Therefore, the range of clinical applications of QSAR analysis is broader than that using only pharmacokinetic information. This study built and provided a detailed analysis of a QSAR predictive model of RR.

## Materials and methods

### Calculation of RR to determine dosage

To provide proper drug treatment for dialysis patients, it is important to determine the amount of drug that must be replenished after hemodialysis.

$$\mathrm{RR }=\frac{(\mathrm{Cpre }-\mathrm{ Cpost})}{\mathrm{Cpre}}$$

$\mathrm{Amount of drug lost during dialysis}=(\mathrm{Cpre }-\mathrm{ Cpost})\times \mathrm{Vd}=\mathrm{RR}\times \mathrm{Cpre}\times \mathrm{V}$ d.Cpre: Plasma concentration of the drug before dialysis.Cpost: Plasma concentration of the drug after dialysis.Vd: Volume of distribution.

The removal rate (RR) of the drug by hemodialysis evaluates the concentration change rate before and after dialysis of the blood concentrations. The amount of drug to replenish can be calculated based on the RR [13].

### Data collection

We investigated the RR from the interview form (IF) in Japan, the Guidebook for Drug in Dialysis Patients volume 3, and both Japan and international literature. IF is a drug manual that corresponds to overseas drug approval information and package inserts. IF is a comprehensive manual containing information submitted by the company to compensate for the insufficient information contained in the package insert of prescription drugs. We did not use RR with blood purification therapy or in vitro. When multiple RRs were found for the same drug, we used the average RR value. We analyzed the certainty and reliability of these data and used the standard deviation (SD) and the root mean square (RMS) of the standard deviation as the variability evaluation score.

SD and RMS were calculated according to the following equations:

$$\mathrm{SD }(\upsigma )=\sqrt{\frac{\sum {(\mathrm{RR observed}-\mathrm{RR average})}^{2}}{\mathrm{N}}}$$

$$\mathrm{RMS}=\sqrt{\frac{\sum_{i=1}^{N}{(\sigma i)}^{2}}{N}}$$

N: Number of RR evaluated.

### Visibility of applicable domains

To better visualize the applicable domains (AD) of 143 drugs better against the descriptors in the dataset, we employed principal component analysis. This is a method for encapsulating related information into summary indices (principal components) that are more readily visualized and analyzed [14]. The chemical space was visualized using a descriptor. To confirm the scientific diversity present in the dataset, descriptors were used to evaluate similarities in chemical structures based on the distances within the chemical space.

### Chemical structure data acquisition and descriptor calculation

The chemical structure of the drug was collected from the PubChem compound database [15] as a simplified molecular-input line-entry system (SMILES). As a numeric representation of the chemical structure, the descriptor was calculated using the chemical computing environment MOE version 2018.0101 (Chemical Computing Group, Inc., Montreal, Canada) [16]. As a pretreatment for descriptor calculation, SMILES strings were treated, desalted, and imparted a partial charge. This was then converted to a 3D structure (4.3.0 0027 30.10.2019) using Corina Classic software (Molecular Networks GmbH, Nürnberg, Germany) [17]. This software has been licensed for the prediction of 3D structures of molecules in large public molecule databases such as PubChem, a data-based commercial 3D molecular model builder with high accuracy and high speed. To optimize the conformation, a force field calculation was then performed (Amber 10 EHT). Based on these chemical structures, we calculated the two-dimensional and three-dimensional descriptors using MOE and Dragon version 7 (Talete srl, Pisani, Milano, Italy) [18]. We excluded descriptors indicating missing values, and descriptors showing complete collinearity (R^2^ = 1) were removed. If the desalted coordination compound did not reflect the pharmacological activity, it became a ligand containing no central metal. Therefore, they were excluded from the dataset. The excluded coordination compounds were carboplatin, gadopentetate, and gadoxetate.

### Construction of predictive models by PLS regression analysis

We collected the RRs of known drugs. After sorting based on RR, the drugs in the dataset were separated randomly into the model construction drug set and the model validation drug set at a ratio of 3:1. Thereafter, we randomly divided these values into the model construction drug set (75%) and model validation drug set (25%). Through the model construction drug set, we performed a PLS regression analysis [7] using RR as the objective variable and chemical structure descriptors as explanatory variables. PLS regression analysis is an improved multiple regression analysis method that prevents multicollinearity by applying a small number of synthetic variables that have been calculated from a number of explanatory variables in the multiple regression analysis. Furthermore, PLS regression analysis can evaluate the importance of the descriptors, which are easier to perform compared to machine-learning methods. We tried machine-learning methods such as random forests (R^2^construction = 0.861, R^2^validation = 0.512) and gradient boosting (R^2^construction = 0.975, R^2^validation = 0.405). These methods were less effective than the PLS method for RR prediction. Among the analyzed algorithms, artificial neural networks provided the best results. PLS method can use a large number of explanatory variables simultaneously. To determine the predictive ability of the model, we used leave-one-out cross-validation [19] as the validation method in the PLS prediction model during construction. The Variable Importance in Projection (VIP) scores was used as an index of the variable importance, and selection was made based on a VIP value of > 0.8. We evaluated the correlation between the predicted and measured values using the coefficient of determination (R^2^), root-mean-square errors (RMSE), and Spearman's correlation coefficient (rs). RMSE and rs were calculated according to the following equations:

$$\mathrm{RMSE}=\sqrt{\frac{\sum {(\mathrm{predicted}-\mathrm{observed})}^{2}}{N}}$$

$$\mathrm{rs}=1-\frac{\sum_{i=1}^{N}{D}^{2}}{N({N}^{2}-1)}$$

N: Number of evaluated drugs.D: Difference in rank between two variables.

We verified the validity of the constructed predictive model using the Durbin–Watson ratio, normal quantile plots, and Cook residual distance (the actual measured value and each of the predicted values).

In these statistical tests, the significance level was set at P < 0.05. The statistical analysis software JMP®Pro 13.2.0 (SAS Institute Inc., Cary, NC, USA) was used in this study.

### Construction of predictive models by artificial neural networks

We attempted to construct an RR estimation model for artificial neural networks (ANNs) using gradient boosting. We randomly divided our values into a model construction drug set (75%) and a model validation drug set (25%). Using the “neural” script in JMP ver.13.2.0 for calculation, we constructed an estimation model from a nonlinear regression of multilayer perception neural networks. A backpropagation training algorithm was employed. The model was validated with prediction of the model validation drug set.

## Results and discussion

### Data collection and preparation

We collected the RR from 143 injection drugs: 99 drugs from the latest IF (March 2020), 25 drugs from the Guidebook for Drug in Dialysis Patients, volume 3, and 19 drugs from various literature sources.

**Fig. 1:**
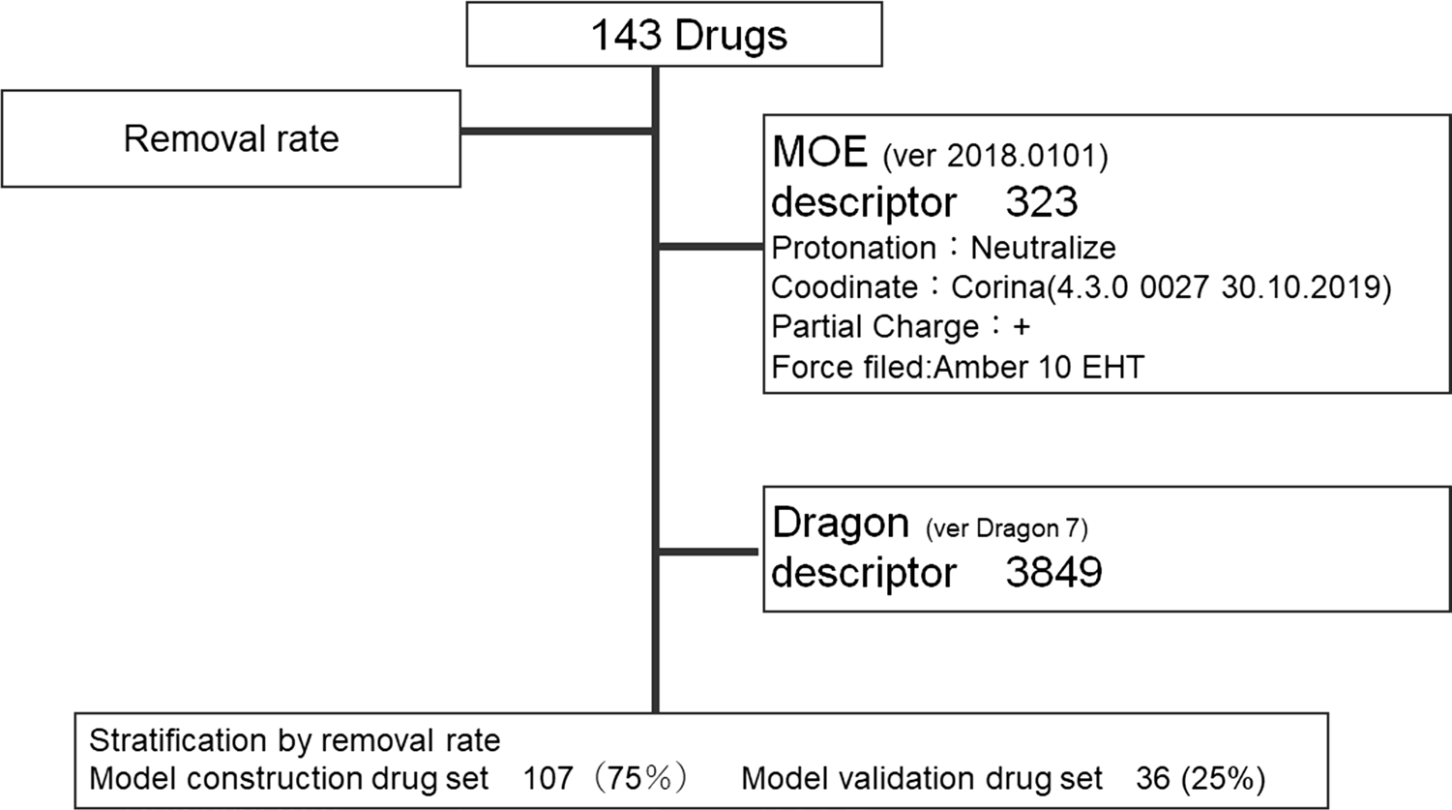
Flow chart of the data table. The RRs of 143 drugs were collected. The objective variable RR and an explanatory variable chemical structure descriptor were constructed as predictive models. The descriptors were calculated as 4,172 pieces. The descriptors between 323 and 3,849 were calculated from MOE and Dragon. The data on 143 drugs were divided into a model construction drug set (107%) and model validation drug set (36%)

The RR of the appropriate item was confirmed manually from an IF. Data of the 143 drugs, including the previously reported RRs, were divided into a model construction drug set (107 drugs, 75%; Table S1) [20–28] and a model validation drug set (36 drugs, 25%; Table S2) [21, 24, 28, 29]. When multiple RRs were found for the same drug, we used the average RR value. The average RR value SD was a maximum of 26.0 and a minimum of 2.6. The RMS of the SD was 9.57. The RMS of the SD indicates overall variability. We calculated 4172 chemical structure descriptors as explanatory variables (Fig. 1). The number of descriptors calculated from the molecular operating environment (MOE) and dragon were 323 and 3849, respectively (Table S3).

**Fig. 2:**
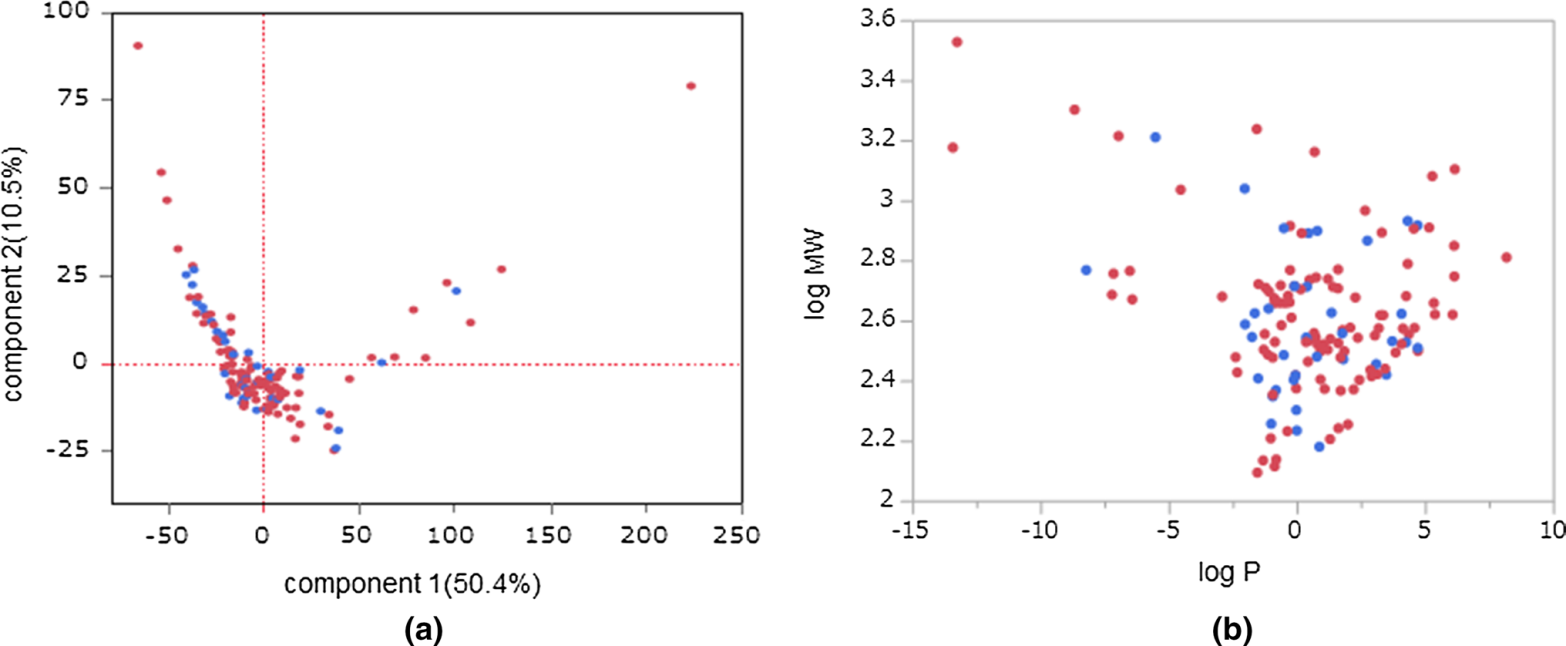
Chemical spaces of 143 compounds. **a** Applicability domain (AD) of 143 compounds. Scatter plot of principal component analysis performed using the descriptors. The horizontal axis represents the first principal component, and the vertical axis represents the second principal component. These percentages are eigenvalue and represent a partition of the total variation in the multivariate sample. Each dot represents a compound; red represents the model construction drug set, and blue represents the model validation drug set. **b** The chemical space is visualized based on the descriptor. The horizontal axis shows the octanol–water partitioning coefficient (log P), and the vertical axis displays the log MW. Each dot represents a compound: red dots represent the model construction drug set, and blue dots represent the model validation drug set

The predictive model was presented in the chemical space model construction drug set. A model can be evaluated locally within its applicability domain (AD) [30, 31]. Figure 2a shows the AD. This scatter plot shows the results of the principal component analysis using descriptors. The horizontal axis represents the first principal component, and the vertical axis represents the second principal component. Moreover, using the log P and MW of the descriptor. The similarity of chemical structures was evaluated for distance within the chemical space to confirm the scientific diversity present using typical physicochemical features. This is shown in the scatter plot (Fig. 2b). We used log P as the log P (o/w) of the chemical structure descriptor, calculated from the MOE. Distributions of the model construction drug set and model validation drug set were well balanced.

### Construction of prediction models using PLS regression analysis

In the PLS regression analysis using the model construction drug set, we performed variable selection using VIP value > 0.8 as a threshold value of variable severity. In the cross-validation, the prediction performance (Q2) was 0.450. The numbers of the selected descriptors and the PLS components were 2,472 (Table S3) and 3 in the RR prediction model, respectively, was constructed. The drug set model was evaluated using leave-one-out cross-validation. The predictive performance of the constructed model was externally validated based on the model validation drug set.

**Fig. 3:**
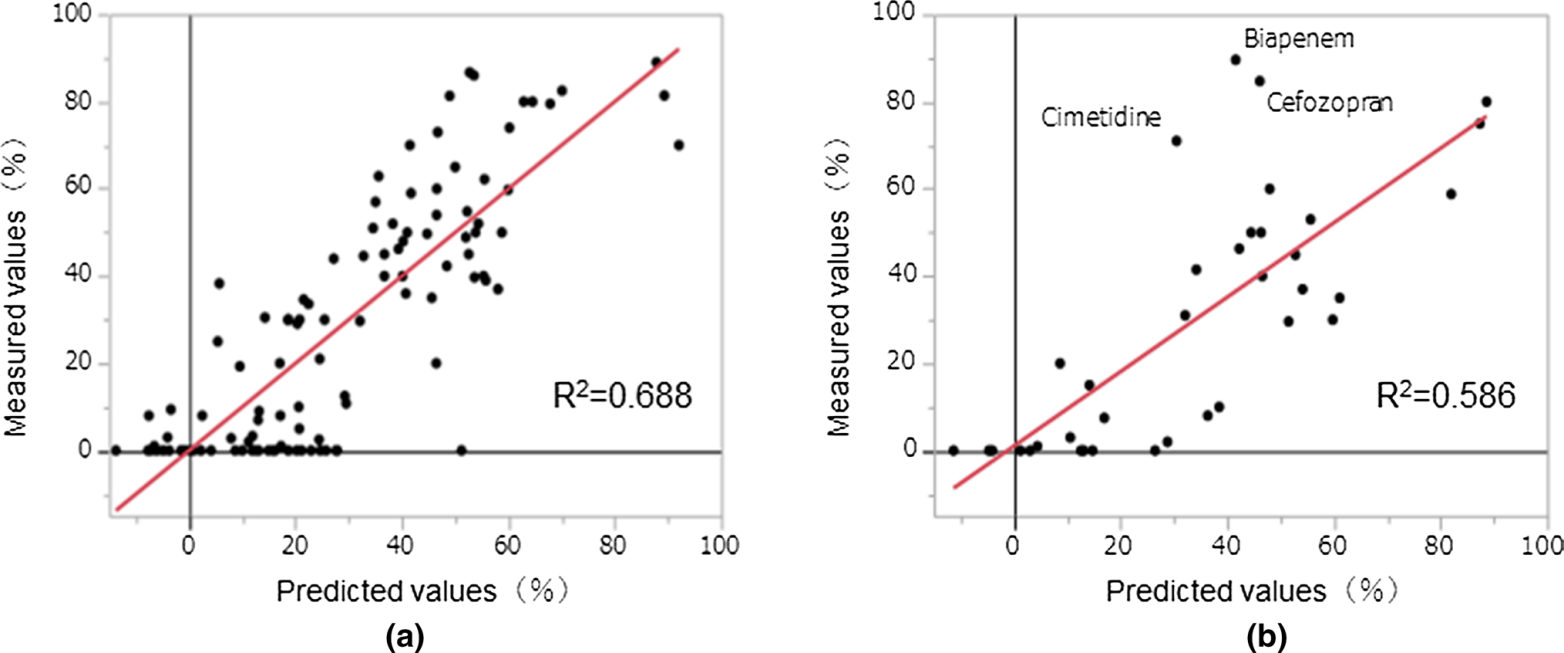
Predicted and measured values for the PLS regression models using data on143 compounds. **a** Scatter plot of the model construction drug set. **b** Scatter plot of the model validation drug set. The horizontal axis depicts RR predicted values, and the vertical axis depicts the RR measured values. Each dot represents a compound. In the model of the validation drug set, the relationship between the measured value and the predicted value for RR was R^2^ = 0.586. Cimetidine, biapenem, and cefozopran were observed to have a relatively large error in the predicted value

Figure 3 shows a scatter plot of the predicted and measured values of the model. In this predictive model, there was a good correlation between the measured RR values in the model construction drug set and the predicted value (R^2^ = 0.688), root-mean-square error of approximation (RMSE) = 15.6, rs = 0.805, Durbin–Watson ratio: 2.19, Cook’s distance of residuals < 0.5) (Fig. 3a).

The generalization performance of the prediction model was evaluated using a model validation drug set 36. In the model validation drug set, the relationship between the measured value and the predicted value for RR was R^2^ = 0.586, RMSE = 19.0, and rs = 0.785 (Fig. 3b). The model shows an expected accuracy comparable to the prediction using previously reported pharmacokinetic parameters. We checked the results using all of the data for the model construction drug set and performed several types of k-fold cross-validation (Table 1). The maximum and minimum Q^2^ values of the k-fold cross-validation were Q^2^ = 0.456 and Q^2^ = 0.380, respectively. The RR values of the model validation drug set were maximum, R^2^ = 0.589, and minimum, R^2^ = 0.582.

**Table 1:** We performed several types of k-fold cross-validation methods to obtain a better idea of the predictability of the model

k-fold	Q2	Construction drug set		Validation drug set	
K10	Q2 = 0.418	R2 = 0.689	RSME = 15.548	R2 = 0.582	RSME = 19.054
K9	Q2 = 0.456	R2 = 0.687	RSME = 15.600	R2 = 0.589	RSME = 18.916
K8	Q2 = 0.435	R2 = 0.687	RSME = 15.549	R2 = 0.584	RSME = 19.016
K7	Q2 = 0.427	R2 = 0.687	RSME = 15.603	R2 = 0.583	RSME = 18.997
K6	Q2 = 0.405	R2 = 0.689	RSME = 15.548	R2 = 0.582	RSME = 19.054
K5	Q2 = 0.380	R2 = 0.689	RSME = 15.548	R2 = 0.582	RSME = 19.054

Considering that the RR data set contains potential errors as shown below, we believe that this study has achieved the most reliable RR prediction accuracy currently available. In future studies, it will be possible to achieve a more accurate prediction by correcting our model using pharmacokinetic parameters.

Patients undergoing dialysis experience delayed excretion, and active metabolites cause adverse side effects. Disopyramide enhances anticholinergic side effects [32], midazolam causes drowsiness [33], and allopurinol causes life-threatening toxicity syndrome [34] in some patients. QSAR analysis is a novel approach to predict RR. If the pharmacokinetic parameters of active metabolites are unknown, QSAR analysis can determine the RR by chemical structure only. Therefore, this method plays an important role in overcoming these challenges.

In the model validation drug set, we observed a relatively large error in the predicted values of cimetidine, biapenem, and cefozopran. The difference between the measured and predicted values is added to the Supplementary Material (Table S4).

Vancomycin is recognized as a rebound phenomenon redistributed in the blood from tissues [35, 36]. The rebound phenomenon is an immediate decrease in the blood concentration of the drug after dialysis, followed by the redistribution of blood levels as the blood rises from subsequent tissue [37]. As a result, a precise QSAR analysis of a drug that produces a rebound phenomenon is difficult. In the model construction drug set, vancomycin had a predicted RR value of 14.25, which was estimated to be lower than the actual value (30.5). The rebound phenomenon reported for vancomycin has also been reported for cimetidine [38]. Due to the rebound phenomenon, the measured RR value of cimetidine was inferred to be high after administration. Due to the rebound phenomenon, some RR values are difficult to set. The time after dialysis at which blood concentrations of the drug were measured was not mentioned in the documents studied. If RR is calculated from the drug concentration in the blood immediately after dialysis, it may be an overestimation if the rebound phenomenon is not taken into account. Most of the measured values provided take the rebound phenomenon into account. However, some of the data in the literature were collected immediately after dialysis and used as the RR [1]. Therefore, we have described only those predicted values that take into account the rebound phenomenon. Therefore, future studies on planned drug administration by hemodialysis should consider the amount of drug removed from the blood immediately after hemodialysis and the amount of drug redistributed from the tissue.

Hemodialysis is also influenced by the flow rate of the blood and dialysate, dialysis time, and hemodialysis conditions, such as the material and surface area of the dialysis membrane. The measured values of biapenem and cefozopran in the model validation drug set were higher than the predicted values. In the calculated dialysis conditions, a polysulfone membrane was used as a dialysis membrane for biapenem. Moreover, a cellulose diacetate membrane, cellulose triacetate membrane, and polysulfone membrane were used for cefozopran. These membranes are classified as high-performance membranes. In the clearance of blood dialysis, ceftriaxone is affected by the type of membrane used [39]. Therefore, the extraction rate of ceftriaxone has been significantly greater in a high-permeability polysulfone membrane than in a hemophane membrane or cuprophane membrane. Biapenem and cefozopran have a high RR using high-performance membranes. Data regarding the description of the drug material for dialysis membranes in pharmaceutical data is lacking. Therefore, the model constructed limits the range of compounds that can be predicted. To further improve prediction accuracy, it is necessary to reduce the bias in the measurement of RR. There is a need to correct the bias of RR measurements using information from several drugs on the rebound phenomenon, the material of the dialysis membrane, the dialysis time, and the flow rate of the dialysate.

### Construction of predictive models by artificial neural networks

Neural networks are powerful tools for the numerical formulation of nonlinear relationships, although it is difficult to determine how much each descriptor contributes to the constructed model. We used the ANN ensemble method with boosting algorithm. In a three-layer ANN consisting of three nodes, 4172 descriptors as explanatory variables provided the best performance. The conditions of hyper-parameters of the ANN were as follows: activation function: hyperbolic tangent, layers: 3, boosting number of models: 28, learning rate: 0.1.

**Fig.4:**
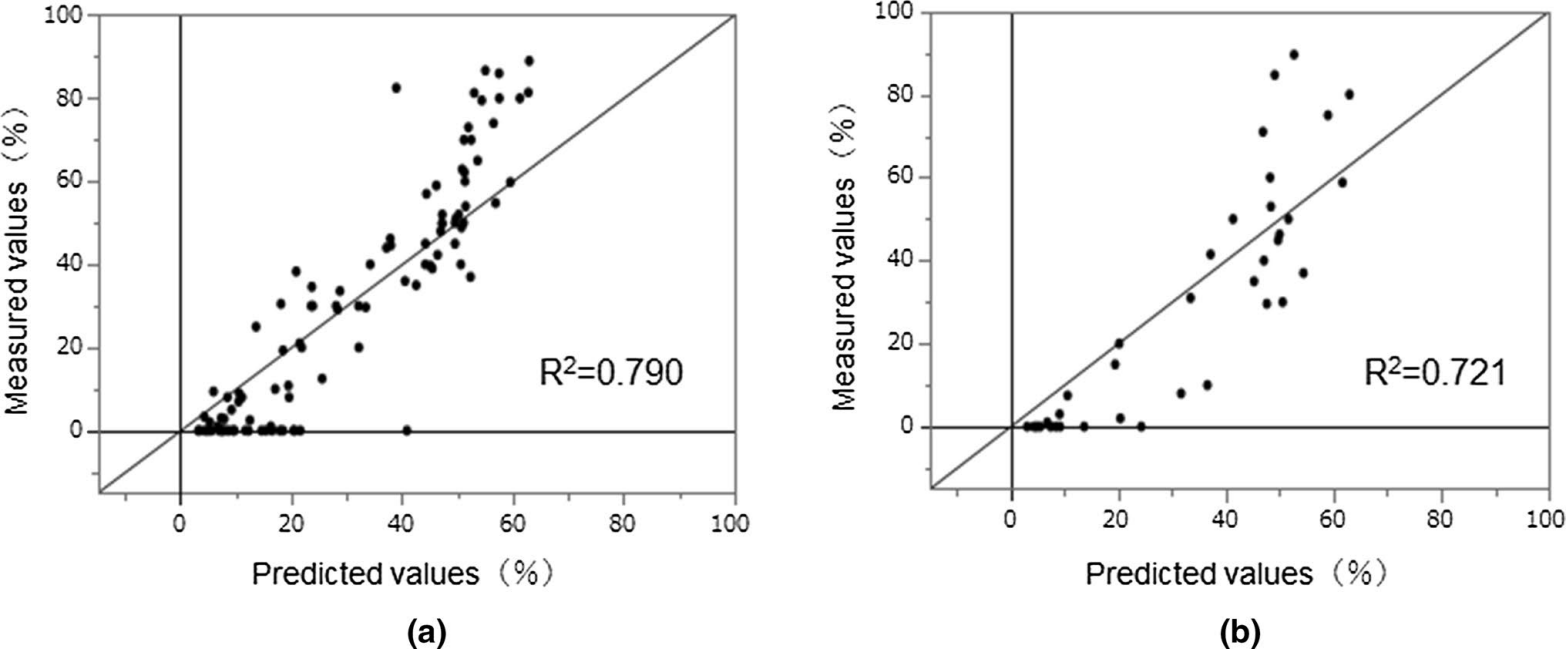
Predicted and measured values for ANN using data on 143 compounds Scatter plots between the measured RR indices and those predicted by the artificial neural network model using data from 143 compounds. **a** Scatter plot of model construction drug set. **b** Scatter plot of model validation drug set. The horizontal axis is RR predicted values, and the vertical axis is RR measured values. Each dot represents a compound. In the model of the verification drugs set, the relationship between the measured values and the predicted values for RR was R^2^ = 0.721.

Using a construction drug set and a validation drug set we were able to successfully create a model that can predict RR with high probability (R^2^ construction = 0.790, RMSE construction = 12.8. R^2^ validation = 0.721, RMSE validation = 14.9) (Fig. 4). Even though the PLS model has relatively low performance, it can be used to interpret the important descriptors, which should be similarly relevant in the ANN model.

### Important contribution descriptor against the RR prediction model

VIP is an indicator of the importance of explanatory variables in modeling the explanatory and objective variables [40]. The VIP of descriptors that have been used for the prediction model is arranged in descending order; it shows the top 10 descriptors.

The chart provides detailed descriptions of each descriptor. B06[N–N]: Presence/absence of N–N at topological distance. R5s + : R maximal autocorrelation of lag/weighted by I-state Q_VSA_FPPOS: fractional positive polar van der Waals surface area PE-OE_VSA_FNEG: Fractional negative van der Waals surface area. Mor23p: weighted by polarizability. PEOE_VSA_FPOS: fractional positive van der Waals surface area ALOGP: Ghose-Crippen octanol–water partition coefficient. AAC: Mean information index of the atomic composition. aICM: Atom information content (mean). This is the entropy of the element distribution in the molecule (including implicit hydrogens, although not lone pair pseudo-atoms). IC0: Information Content Index (neighborhood symmetry of the 0-order). The descriptor that contributed the most to each PLS component is shown. The coefficient of determination for ATS1v was 0.934 for PLS component 1. The coefficient of determination of vsurf_CW4 was 0.613 for PLS component 2. The coefficient of determination for HATS8s was 0.372 for PLS component 3. This provides detailed information regarding each descriptor. ATS1v: Broto–Moreau autocorrelation of lag (log function). vsurf_CW4: capacity factor HATS8s: leverage-weighted autocorrelation of the lag meaning of PLS components and descriptors. The PLS component 1 represents the van der Waals volume. PLS component 2 represents the utility of the pharmacokinetic property prediction. The PLS component 3 represents the I-state.

Table 2 lists the top 10 contributing descriptors for the prediction model. VIP is an indicator of the importance of the explanatory variables in the prediction model. The importance of the descriptor was defined by the VIP. The VIPs of descriptors that have been used for the prediction model are arranged in descending order; the top 10 descriptors are shown, with the VIP and relevant characteristics given for each. The descriptors and SMILES for all compounds used in the prediction model are described in the supplementary data (Table S4). To construct an RR prediction model using fewer descriptors, we reduced the number of molecular descriptors from 2472 to 10. Pleasingly, the RR prediction model with the top 10 contributing descriptors for the prediction model exhibited similar or better scores (R^2^ = 0.608 and RMSE = 18.1) than the model using 2472 descriptors in the model validation drug set. The descriptors do not contribute equally. The importance is represented by the VIP level; the top 10 descriptors showed a particularly important influence on the major characteristics. Important descriptors were associated with topological shape, electric state, and lipophilicity. log P shows that lipophilicity aptly explains many of the features of the drug, such as the PBR [41]. Urata et al. [11] reported that PBR is an important pharmacokinetic parameter in RR prediction and that PBR is high in drugs with a low RR. In this study, knowledge of the importance of log P in the QSAR model for RR supports the results of a previous study. Urata et al. [11] established a multiple regression model for 90 drugs and evaluated a predictive model using 17 drugs. They reported R2 = 0.64, using molecular weight and three pharmacokinetic parameters. This is currently the only model available for predicting RR. The model in this study uses 143 drugs for model construction and evaluation. Notably, this is the largest dataset for RR prediction reported to date. PLS regression analysis is capable of constructing highly accurate prediction models even when using a large number of explanatory variables. It also has the advantage of being highly explanatory for the selected variables. Despite this, neural networks provided the best results (R2 = 0.721 RMSE = 14.936). These were better than any previously reported by other RR prediction methods. RR prediction by QSAR analysis has the advantage that it can be performed solely on chemical structure instead of pharmacokinetic parameters as these are not always available. The RR prediction model described herein is expected to be valuable in clinical settings.

**Table 2:** Top 10 descriptors made a significant contribution to the prediction of the PLS regression analysis models

	Descriptor	VIP	Meaning
1	B06[N–N]	2.264	Topological shape
2	R5s +	2.172	3D shape and size
3	Q_VSA_FPPOS	2.072	Topological shape and electric state
4	Mor23p	1.978	3D shape and electric state
5	PEOE_VSA_FNEG	1.977	Topological shape and electric state
6	PEOE_VSA_FPOS	1.977	Topological shape and electric state
7	ALOGP	1.902	Lipophilicity
8	AAC	1.894	Atomic composition
9	IC0	1.894	Symmetry
10	a_ICM	1.894	Topological shape and lipophilicity

### Molecular structure in the physiological pH conditions

**Fig. 5:**
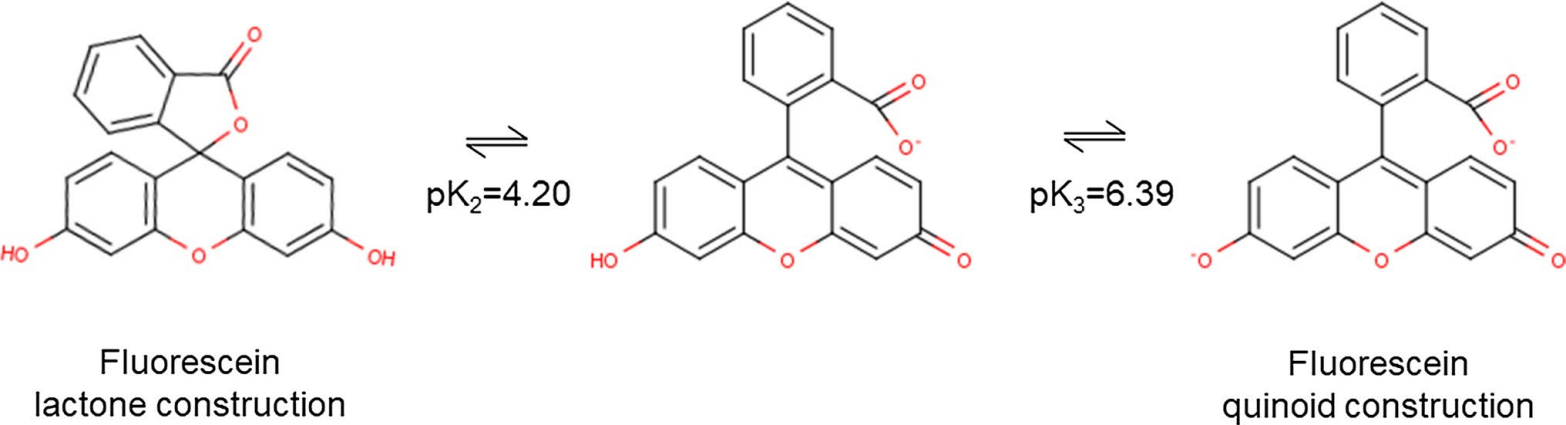
Fluorescein molecular structure under physiological pH conditions. Fluorescein exhibits lactone structures under acidic conditions. The lactone structure exhibited a quinoid structure by ring-opening the lactone structure under physiological pH conditions

When constructing a predictive model using the lactone structure of fluorescein, the RR predictive value deviated significantly from the measured value. To examine this error, structural changes in fluorescein were considered. Fluorescein changes its molecular structure according to pH conditions. Therefore, we reconstructed the prediction model using the quinoid structure, which has a high probability of existence at pH of blood maintained in the body. Usually the body maintains the pH of blood close to 7.40. Fluorescein with triphenylmethane structures are known to exhibit lactone structures and quinoid structures, respectively, under acidic and neutral conditions (Fig. 5) [42]. The lactone ring affected the accuracy of the prediction model. Of the drugs in this drug dataset, fluorescein was the only drug that formed a lactone ring. In the above prediction model, fluorescein was treated as a quinoid structure under physiological conditions.

**Fig. 6:**
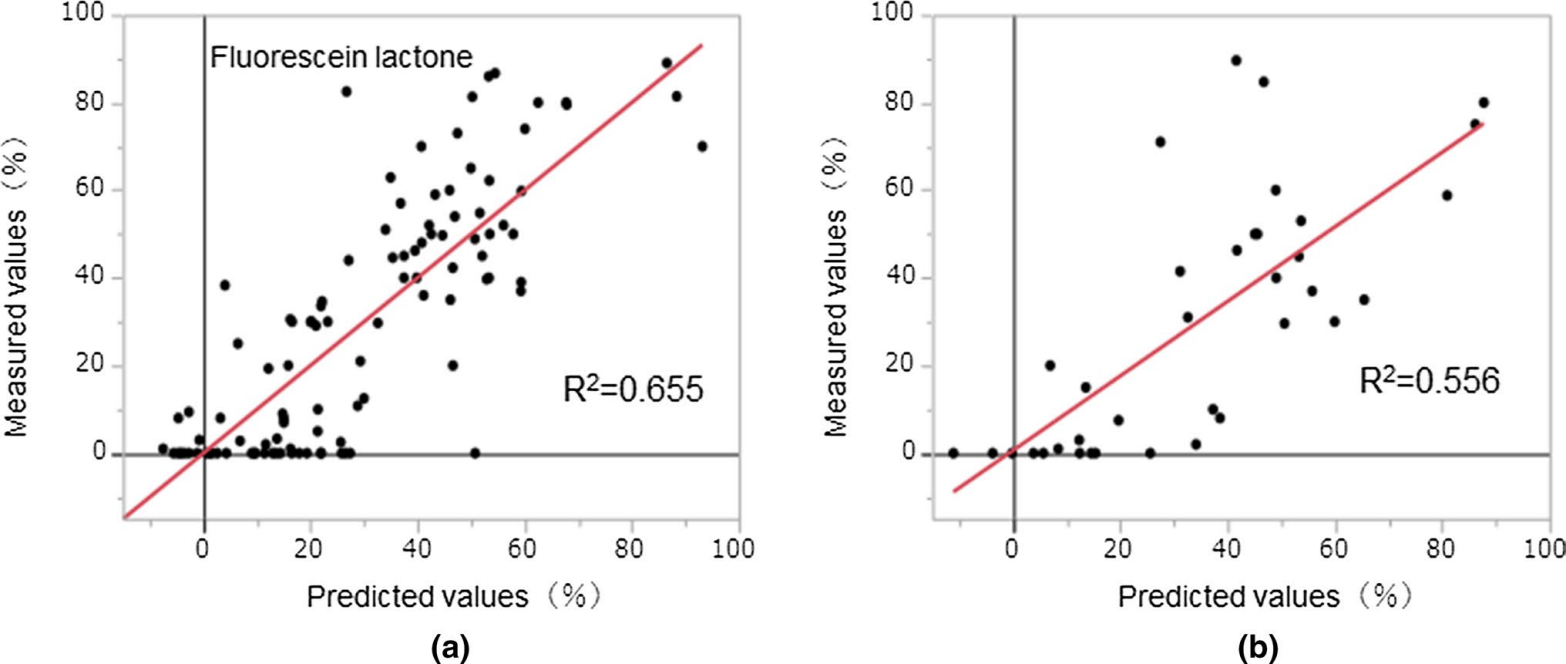
Predicted and measured values for the PLS regression models based on a descriptor of the fluorescein lactone structure. **a** Scatter plot of the model construction drug set. When building the predictive model using the lactone structure of fluorescein, the RR predictive value deviated significantly from the measured value. To consider this error, structural changes in the fluorescein were verified. **b** Scatter plot of the model validation drug set. The horizontal axis shows RR predicted values, and the vertical axis shows RR measured values. Each dot represents a compound. The fluorescein lactone structural descriptor was used to develop a predictive model. In the verified drugs set model, the relationship between the measured value and the predicted value for RR was R^2^ = 0.556

In contrast, the fluorescein descriptors in the model construction drug set were calculated from the lactone structure and used to construct the predictive model, as in the case of the quinoid structure. The statistics of the prediction model in the model construction drug set indicated the following values: R^2^ = 0.655, RMSE = 16.4, and rs = 0.793 (Fig. 6a). In the fluorescein lactone structure, the RR predictive value (26.7) deviated significantly from the measured value (82.5). The RR predictive value in the model validation drug set was R^2^ = 0.556, RMSE = 19.7, and rs = 0.766. Figure 6b shows a scatter plot of RR in the model validation drug set. In the prediction model, fluorescein was constructed from the lactone structure. These results indicate that the inadequate lactone structure of fluorescein affects the accuracy of the prediction model. Fluorescein is used in diagnostic fluorescein angiography or angioscopy of the retina and iris vasculature. When using this drug in patients with renal failure or excretion, blood concentrations must be monitored for elevation. Dialysis is an important route for clearance in patients undergoing dialysis. The determination of the RR is clinically important. To predict the RR of drugs with similar chemical characteristics, such as fluorescein, the prediction model must include information on combining fluorescein chemical structure and the RR. Furthermore, obtaining useful information on a compound possessing a lactone by analyzing the fluorescein composition. When predicting the RR using the current prediction model, the chemical structure of the drug under physiological pH is considered essential for obtaining a prediction result with higher accuracy.

## Limitations

This dataset, which used leave-one-out cross-validation, was small. The ideal validation methods are the leave-one-cluster out [43] and nested cross-validation [44], which prevent overfitting. It may be possible to construct a more reliable predictive model using these verification methods when the number of samples is increased.

## Conclusion

This study used QSAR analysis to successfully construct a prediction model of the drug RR in dialysis, with accuracy comparable to that of models using pharmacokinetic parameters. The RR, as predicted by the QSAR analysis, may be determined using only the chemical structure of the drug. Considering that the RR data set contains potential errors, we believe that this study has achieved the most reliable RR prediction accuracy currently available. Such a model is expected to be applied to active metabolites and dangerous drugs, which have limited information on pharmacokinetic parameters.

## Supplementary Information

Below is the link to the electronic supplementary material.

Table S_1_ The removal rate of drugs. (Model construction drug set). Data of the 143 drugs, including the previously reported RRs, were divided into a model construction drug set (107 drugs, 75%). Table S_2_ The removal rate of drugs. (Model validation drug set). Data of the 143 drugs, including the previously reported RRs, were divided into a model validation drug set (36 drugs, 25%). Table S_3_ Reporting the number of descriptors calculated from MOE and Dragon were 323 and 3849, respectively. The selected descriptor was 2,472 for constructing the prediction models. Table S_4_ Reporting the descriptors and SMILES, the predicted values for all compounds. The difference between the measured and predicted values in the prediction model is shown. (XLSX 3472 kb)
